# Entomological survey of *Dirofilaria* spp. in Sardinia (Italy): molecular detection and mosquito species distribution

**DOI:** 10.1186/s13071-026-07425-x

**Published:** 2026-05-08

**Authors:** Lia Cavallo, Eleonora Perugini, Francesca Nonnis, Cinzia Pasini, Gabriele Tosciri, Pamela Zeinoun, Serena Cavallero, Claudia Tamponi, Antonio Scala, Antonio Varcasia, Simona Gabrielli, Marco Pombi

**Affiliations:** 1 Department of Veterinary Medicine, University of Sassari, Via Vienna 2, 07100 Sassari, Italy; 2 Department of Public Health and Infectious Diseases, Sapienza University of Rome, Piazzale Aldo Moro 5, 00185 Rome, Italy; 3 Service for Hygiene of Production and Marketing of Food of Animal Origin (SIAOA), ASL Sulcis Iglesiente, Via Gorizia SNC, 09016 Iglesias, Italy; 4 Animal Health Service, ASL Lanusei, Viale Don Bosco 28, 08045 Lanusei, Italy; 5 Department of Public Health and Infectious Diseases, Sapienza University of Rome, laboratory affiliated to Istituto Pasteur Italia-Fondazione Cenci Bolognetti, Piazzale Aldo Moro 5, 00185 Rome, Italy

**Keywords:** *Dirofilaria immitis*, *Dirofilaria repens*, Vectors, Entomological surveillance

## Abstract

**Background:**

Dirofilariosis, caused by *Dirofilaria immitis* and *Dirofilaria repens*, is expanding across Europe because of climate change, raising veterinary and zoonotic concerns. Among over 70 mosquito species considered to be implicated in their transmission, *Culex pipiens* and *Aedes albopictus* are recognized as major vectors. In Sardinia, where canine dirofilariosis is endemic, entomological information on *Dirofilaria* circulation was previously lacking. This study investigated mosquito species composition and assessed the presence of *D. immitis* and *D. repens* across five sites on the island.

**Methods:**

Mosquitoes were collected monthly from August 2022 to October 2023 using BG-Sentinel and CDC light traps. Specimens were morphologically and molecularly identified and screened for *Dirofilaria* DNA.

**Results:**

A total of 1219 mosquitoes were collected, including 945 females belonging to 13 species. The most abundant were *Aedes caspius* (31.4%), *Aedes detritus* (28.7%), *Culex pipiens* (19.5%), and *Aedes albopictus* (16.6%). Notably, among minor species, the presence of *Culex perexiguus* in Italy was confirmed for the first time by molecular analysis. *Dirofilaria repens* and *D. immitis* DNA were detected in 1.8% and 1.0% of mosquitoes, respectively, with *C. pipiens* and *Ae. albopictus* as the main vectors. Other species, including *Ae. caspius*, *Ae. detritus*, *C. tarsalis*, and *C. perexiguus*, also tested positive, suggesting a potential role in transmission.

**Conclusions:**

This study provides the first entomological report of *Dirofilaria* circulation in Sardinia, revealing a complex vector community and underscoring the need for continuous surveillance of dirofilariosis risk in the Mediterranean region.

**Graphical Abstract:**

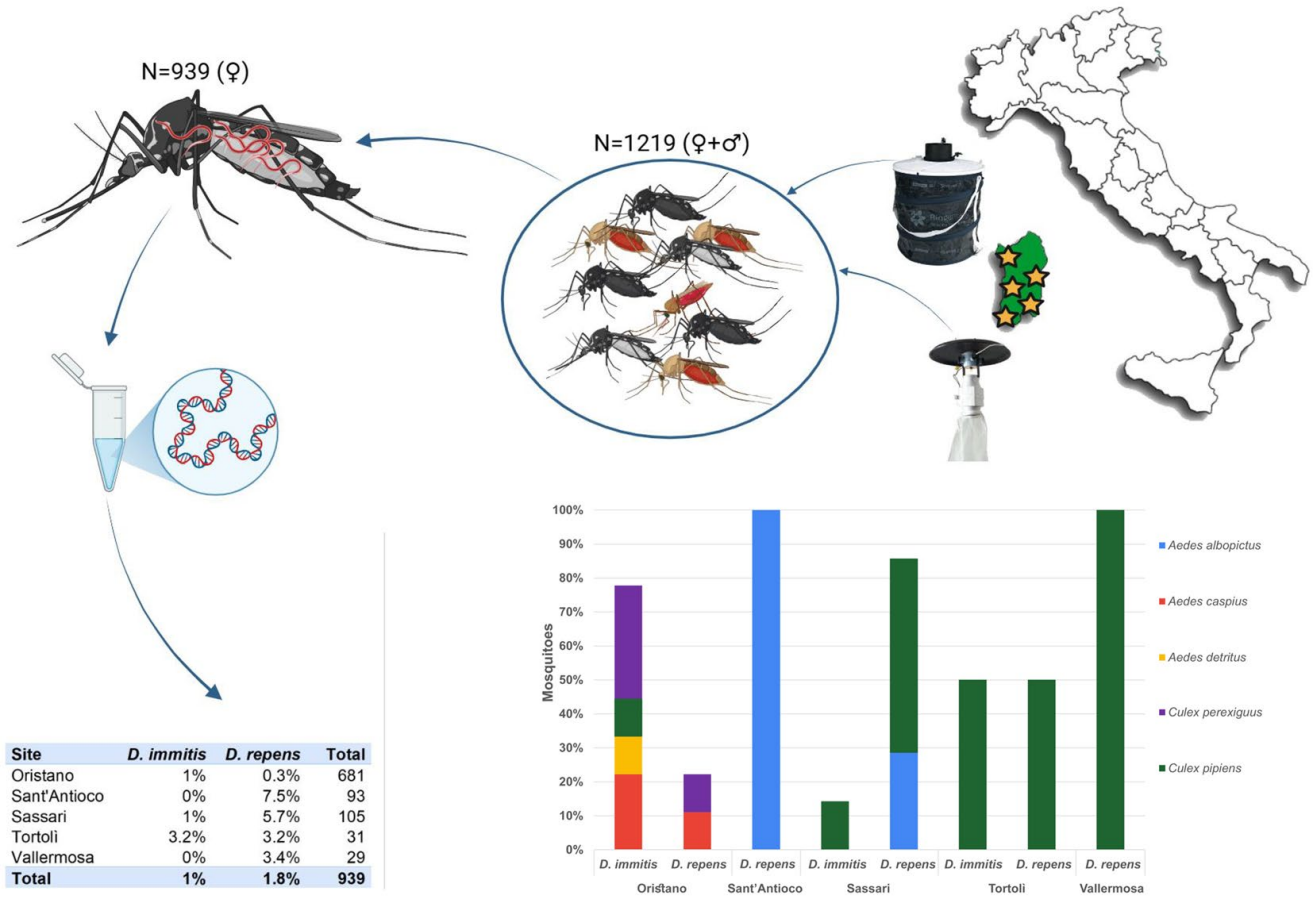

## Background

In recent years, anthropogenic changes, such as climate change, urbanization, and environmental pollution, have significantly influenced mosquito distribution, promoting the introduction and spread of invasive and native species beyond their original ranges [1–4]. Therefore, the shifts in mosquito fauna have altered the epidemiology of certain vector-borne diseases (VBDs). For example, in Europe, the European Centre for Disease Prevention and Control (ECDC) has reported a rise in locally acquired dengue and chikungunya cases in previously unaffected EU/EEA regions, alongside expanded occurrences of West Nile virus, linked to broader mosquito distribution [5]. Beyond arboviruses, another VBD of public health importance, experiencing changes in its distribution range in Europe, is dirofilariasis. Both *Dirofilaria immitis* and *Dirofilaria repens* (Spirurida, Onchocercidae) are present in the continent as causative agents, respectively, of heartworm disease (HWD) and subcutaneous filariosis in wild and domestic canids [6–8]. In recent decades, the epidemiological picture of canine dirofilariosis has changed as a consequence of climate change [9, 10]. Originally considered endemic in Mediterranean regions, *Dirofilaria repens* has progressively expanded into north-eastern and eastern Europe. Similarly, *D. immitis* has become increasingly prevalent in southern regions [11, 12].

In addition to their veterinary relevance, these parasites can also infect humans, causing human pulmonary and subcutaneous/ocular dirofilariosis worldwide [13]. Historically considered an incidental infection, human dirofilariosis has recently undergone a significant epidemiological shift, with the rising incidence of certain clinical forms leading to its recognition as an emerging disease in Europe [14, 15]. Up to 70 mosquito species, belonging to the genera *Aedes*, *Anopheles*, and *Culex*, are implicated in the transmission of *D. immitis* and *D. repens*, with *Culex pipiens* and *Aedes albopictus* recognized as major vectors in Europe [8, 9, 16].

Sardinia, a Mediterranean Italian island in the south of Italy, is characterized by high mosquito species diversity, comparable to that of the mainland, and by the stable presence of the invasive species *Ae. albopictus*, with the first records dating back to the mid-1990s and early 2000s [17–19].

A recent study in Sardinia reported a prevalence of 15.2% for *Dirofilaria* spp. in dogs (9.9% *D. immitis*, 5.5% *D. repens*), confirming the island as endemic [12] and raising concern about zoonotic transmission, in line with human autochthonous cases reported in other endemic Italian regions [20, 21]. These findings highlight the importance of investigating local mosquito populations, because competent vectors are essential for parasite transmission, in a One Health perspective.

Despite the high prevalence observed in dogs in Sardinia, to date, no study has investigated the infection in humans or the presence of *Dirofilaria* spp. within the local mosquito population. This study aims to identify mosquito species composition in selected areas of Sardinia to detect potential vectors of *Dirofilaria* spp. by assessing parasite occurrence through molecular detection in different species and thereby infer their possible role in local transmission.

## Methods

### Mosquito sampling and processing

From August 2022 to October 2023, mosquitoes were captured from five peri-urban sites across Sardinia Island: Sassari (40.7088548, 8.5527459, site SS; province of Sassari), Tortolì (39.9596520, 9.6549270, site TO; province of Nuoro), Oristano (39.9396860, 8.5834800, site OR; province of Oristano), Vallermosa (39.3731240, 8.8011590, site VA, province of Cagliari), and Sant’Antioco (39.1005126, 8.3998127, site SA, province of Cagliari). These locations were chosen based on their geographical distribution to represent the environmental and climatic variability of the region. Sassari is an urbanized area in the northwest, characterized by a mild, windy Mediterranean climate featuring cool, wet winters and hot, dry summers. Tortolì is a rural area with moderate human presence, located on the East Coast, with a humid Mediterranean climate, moderate temperatures, and sheltered conditions. Natural wetlands and floodplains with significant agricultural activity, including rice fields, characterize the moderately anthropized area of Oristano; it is on the west-central coast, with hot, humid summers and mild winters. Vallermosa is a predominantly rural area with scattered semi-natural landscapes; it is in the south, with a warmer, drier Mediterranean climate marked by hotter summers and cooler winters. Sant’Antioco is a moderately urbanized Southern Island with a warm maritime Mediterranean climate, hot summers, and mild winters.

Mosquito captures were performed using two different types of traps: BG-Sentinel 2 baited with BG-lure (hereafter BG) and CDC light traps (model IMT, PeP, San Giuliano Milanese, Italy, hereafter CDC). In each site, 24 h monthly samplings were conducted using two BG and two CDC light traps. To avoid reciprocal interference, traps at each site were placed at a minimum distance of 20 m from each other. After the 24 h trapping period, the samples were transported to the Parasitology Laboratory at the Department of Veterinary Medicine, University of Sassari, in polystyrene containers filled with dry ice.

Mosquitoes were morphologically identified to the species level [22] and then sorted by species, sex, and gonotrophic stage and stored in silica gel for further molecular analyses.

### Molecular detection of *Dirofilaria* spp.

Genomic DNA was extracted from either a single specimen or a pool of 2–6 mosquitoes, using the Quick-DNA™ Miniprep Kit (Zymo Research, USA). The extracted DNA was subjected to PCR amplification targeting the 5S rRNA region, following the protocol described by Xie et al. (1994) [23]. This reaction typically yields an amplification product of approximately 400 base pairs (bp) for most filarial species. In the case of *D. repens*, an additional fragment of around 350 bp is also observed [24]. *Dirofilaria immitis* identification was further confirmed using species-specific primers, as previously described [25]. In the presence of *Dirofilaria* DNA, the pool tested was considered positive for one mosquito only to avoid overestimation of positivity rate.

### Molecular identification of mosquito species

For the mosquitoes with damaged morphological characters but that tested positive for *Dirofilaria*, the species was confirmed through sequencing. DNA was amplified using universal primers targeting a fragment of the mitochondrial cytochrome c oxidase subunit I (COI) gene, commonly used for species identification in metazoan invertebrates [26].

PCR products were sequenced bidirectionally using the same primers and BigDye Terminator v3.1 chemistry on a 3130 Genetic Analyzer (Applied Biosystems, Foster City, CA, USA). Nucleotide sequences were edited, aligned using ClustalW [27], and analysed using BioEdit. Then, they were compared with publicly available sequences in GenBank using the Basic Local Alignment Search Tool (BLAST; http://blast.ncbi.nlm.nih.gov/Blast.cgi).

### Statistical analysis

Species diversity per each sampling site was quantified using the Shannon-Wiener Diversity Index (*H*). The index was calculated based on the site-aggregated abundance of each species using the formula:

$$H=-{\sum}_{i=1}^{S}{p}_{i}ln({p}_{i})$$

where *S* is the total number of species, and *p*_*i*_ is the proportional abundance of the *i*—th species (i.e., the number of individuals of species *i* divided by the total number of individuals observed at that site).

The 95% confidence intervals (CI) of *H* values were determined using a non-parametric bootstrap procedure using mosquito count as the sampling unit (2000 resamplings) and calculating the H index for each resampling. The 95% CI was calculated using the 2.5th and 97.5th percentiles of the 2000 bootstrapped H values as the lower and upper bounds of the interval, respectively.

All statistical analyses were performed using R software [28], utilizing the vegan package for diversity calculation [29] and custom scripts for the bootstrap procedure.

## Results

### Species composition and population dynamics

A total sampling effort of 60 days was conducted in study areas (from 29 August 2022 to 31 October 2023), for a total of 240 trap samplings. Of the 1219 mosquitoes collected in the five areas sampled (BG 36.3%, CDC light 63.7%), 945 specimens were properly identified as females and 274 as males. Among females, 95.3% were unfed, 3.3% engorged, and 1.4% gravid. The 75.2% female specimens morphologically identified at the species level indicated a total of 13 species collected, most of them belonging to the subfamily Culicinae (Table 1). Only one mosquito, identified as *Anopheles plumbeus*, an unfed female, belonged to the Anophelinae subfamily and was collected in Oristano. Overall, *Aedes caspius* was the most abundant species (29.7%), followed by *Ae. detritus* (27.8%), *Culex pipiens* (19.8%), and *Ae. albopictus* (16.5%) among all mosquitoes properly identified by species (*N* = 711; Table 1). Notably, among the minor species collected, 12 individuals (grouped in seven pools) were molecularly identified as *Culex perexiguus*, a species not conclusively reported in Italy before [17, 30–32] (accession no. PX525566). The sequences obtained in this study exhibited a percentage identity of 98.75%–99.70% with those deposited in GenBank.

**Table 1:** Total of female mosquitoes identified at the species and genus level, divided by sampling site (ND: not determined)

Species	Oristano	Sant’Antioco	Sassari	Tortolì	Vallermosa	Total
	(*N*)	(*N*)	(*N*)	(*N*)	(*N*)	
*Aedes caspius*	219	1	0	1	0	221
*Aedes detritus*	191	3	0	0	4	198
*Culex pipiens*	57	9	52	14	9	141
*Aedes albopictus*	19	61	27	7	3	117
*Aedes mariae*	6	2	0	0	1	9
*Aedes rusticus*	5	0	0	0	0	5
*Aedes geniculatus*	1	2	0	0	0	3
*Culex perexiguus*	12	0	0	0	0	12
*Culex theileri*	0	0	0	0	2	2
*Anopheles plumbeus*	1	0	0	0	0	1
*Culex modestus*	0	1	0	0	0	1
*Culiseta annulata*	0	1	0	0	0	1
ND	172	15	26	11	10	234
Total	511	80	79	22	19	711

At the site level (Table 1), *Ae. caspius* and *Ae. detritus* were the dominant species collected in Oristano (219/511, 42.9% and 191/511, 37.4%, respectively), *Ae. albopictus* was the most abundant in Sant’Antioco (61/80, 76.3%), while *C. pipiens* accounted for most of the collections in Sassari (52/79, 65.8%), Tortolì (14/22, 63.6%), and Vallermosa (9/19, 47.4%).

The Shannon index (H) for the different sites (Table 2) indicated Vallermosa as the site with the most diverse species community, followed by Oristano. Sant’Antioco showed intermediate species diversity, while Sassari and Tortolì were characterized by the lowest species diversity observed in the study area (Table 2).

**Table 2:** Species diversity indices at sampling sites (95% CI: confidence interval)

Site	Shannon index	Lower 95% CI	Upper 95% CI
Oristano	0.82	0.78	0.85
Sant’Antioco	0.69	0.65	0.73
Sassari	0.53	0.48	0.58
Tortolì	0.54	0.49	0.59

**Fig. 1:**
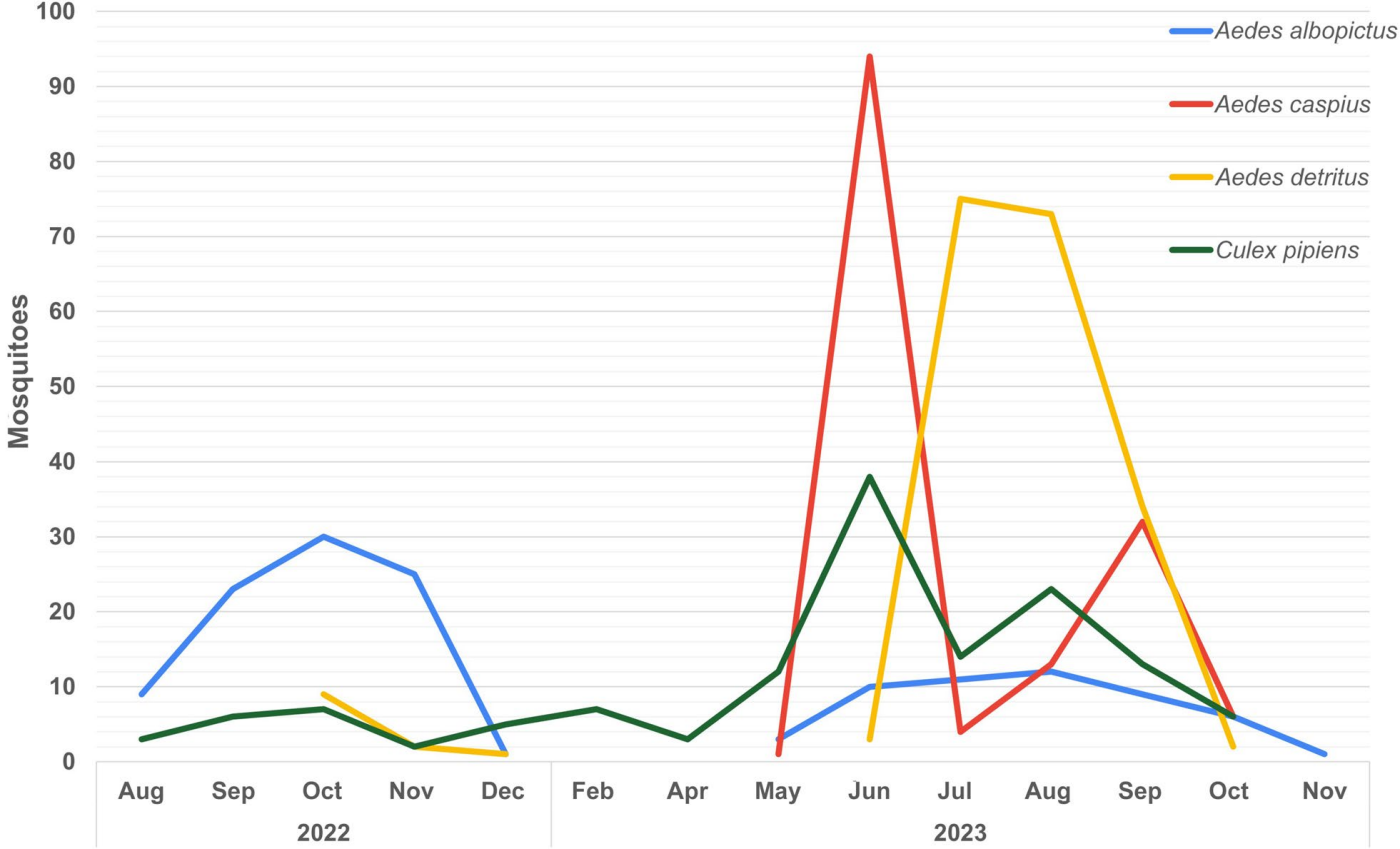
Species population dynamics over the 14-month sampling period

The most mosquitoes were captured between May and October, although different population dynamics were observed among the most abundant vector species (Fig. 1). *Aedes caspius* exhibited bimodal activity, peaking in June and September, while *Ae. detritus* maintained consistently high densities from June through September. In contrast to these species, *Ae. albopictus* and *C. pipiens* showed a broader seasonal distribution, with activity extending beyond the May–October period. Notably, *C. pipiens* was present year-round, with peak abundances in June and August, whereas *Ae. albopictus* displayed an extended activity window from May to December.

### *Dirofilaria* spp. abundance

Among the 945 female mosquitoes collected, 939 were grouped in 389 pools (1—6 mosquitoes) and molecularly analysed for *Dirofilaria* presence. *Dirofilaria repens* and *D. immitis* DNA was identified in 1.8% (17/939) and 1% (9/939) of the mosquitoes, respectively (Table 3). All positive mosquitoes showing damaged morphological characters were confirmed at the species level by sequencing. All the sites analysed were positive for *D. repens.* The highest positivity rate for this species was observed in Sant’Antioco (7.5%; 7/93), followed by Sassari (5.7%; 6/105), Vallermosa (3.4%; 1/29), Tortolì (3.2%; 1/31), and Oristano (0.3%; 2/681). Concerning *D. immitis*, three sites tested positive, with the highest rate recorded in Tortolì (3.2%; 1/31), followed by Sassari (1%; 1/105) and Oristano (1%; 7/681) (Table 3).

**Table 3:** Positivity rate for *Dirofilaria immitis* and *Dirofilaria repens* at the sampling sites

Site	*D. immitis *(%)	*D. repens *(%)	Total tested specimens	Positive pools/total pools tested
Oristano	1.0	0.3	681	9/234
Sant'Antioco	0	7.5	93	7/58
Sassari	1.0	5.7	105	7/52
Tortolì	3.2	3.2	31	2/24
Vallermosa	0	3.4	29	1/25
Total	1.0	1.8	939	26/390

The positivity rate is calculated from positive pools, assuming only one positive mosquito per pool

Among all mosquito species that resulted positive for *D. repens* (*N* = 17), the relative contribution of each species is as follows: 52.9% *Ae. albopictus*, 35.3% *C. pipiens*, 5.9% *Ae. caspius,*, and 5.9% *C. perexiguus*; for *D. immitis* (*N* = 9): 33.3% *C. perexiguus*, 33.3% *C. pipiens*, 22.2% *Ae. caspius*, and 11.1% *Ae. detritus*.

**Fig. 2:**
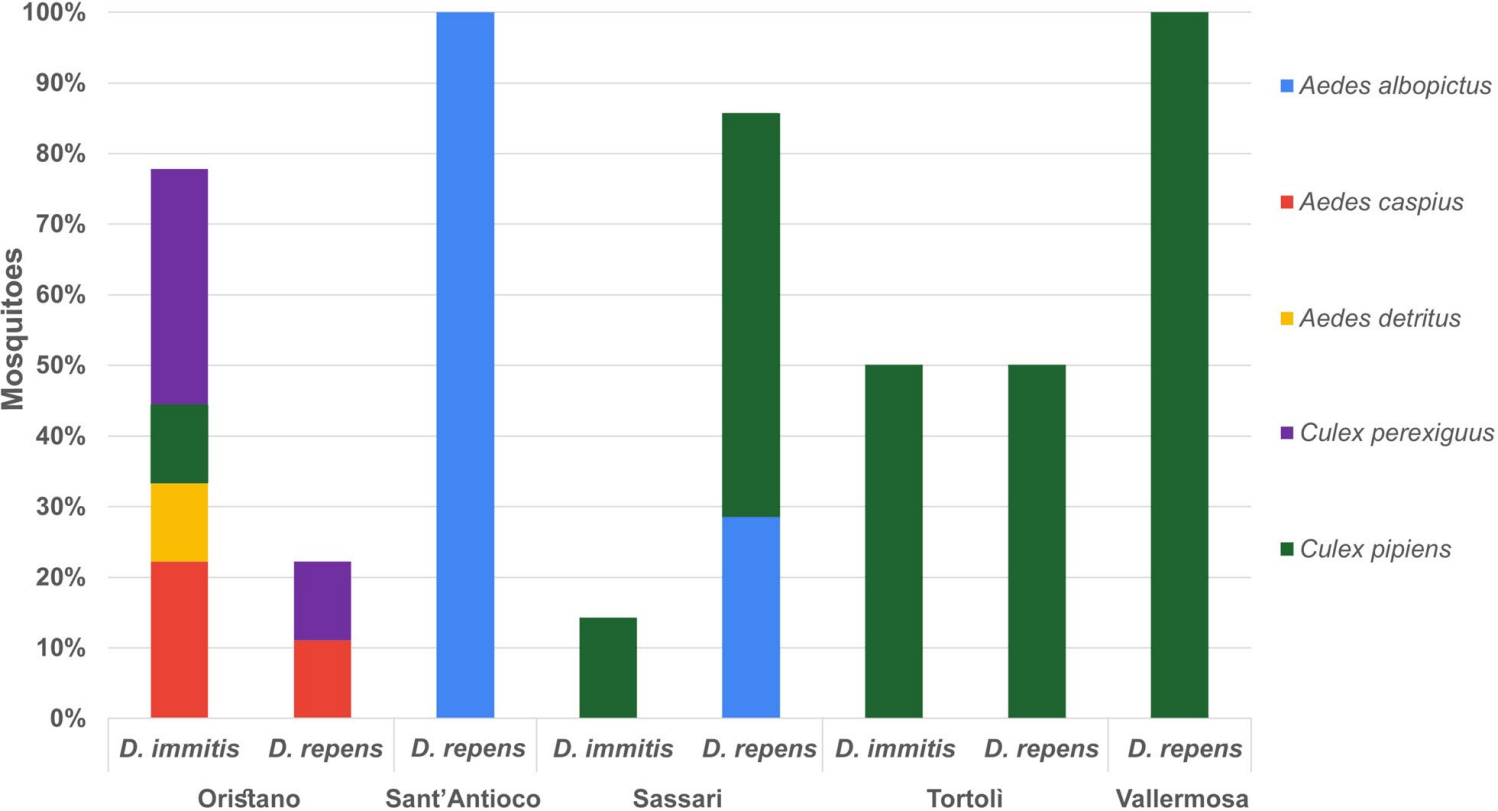
Relative proportion of *Dirofilaria immitis* and *Dirofilaria repens* among different sites, with the relative contribution of mosquito species

*Dirofilaria*-positive specimens were distributed differently across sites, with varying relative contributions of each species. In particular, Oristano was the only site with more than two species infected (Fig. 2).

The positivity rate per each species (Table 4) is as follows: *Ae. albopictus* exhibited the highest positivity for *D. repens* (7.8%; *N* = 116), while no mosquitoes of this species tested positive for *D. immitis*. *Aedes detritus* (*N* = 198) only proved positive for *D. immitis* (0.5%). The only species found infected with both parasites were *Ae. caspius* (*D. immitis* 0.9%; *D. repens* 0.5%; *N* = 220), *C. pipiens* (*D. immitis* 2.1%; *D. repens* 4.3%; *N* = 141), and *C. perexiguus* (*D. immitis* 25%; *D. repens* 8.3%; *N* = 12; Table 4). For the mosquitoes that tested positive and for which morphological species identification was doubtful, requiring confirmation through sequencing, the COI sequences have been deposited in GenBank under accession nos. PX963966, PX963967, PX963968, PX963969, PX963970, PX963971, and PX963972.

**Table 4:** Total number of mosquitoes captured, number of positive specimens, and positivity rates according to mosquito species and sampling site

Species	Oristano			Sant'Antioco		Sassari			Tortolì			Vallermosa		All sites			
	*Dirofilaria immitis*	*D. repens*	Total	*D. repens*	Total	*D. immitis*	*D. repens*	Total	*D. immitis*	*D. repens*	Total	*D. repens*	Total	*D. immitis *(%)	*D. repens *(%)	Total analysed	N positive pools/total pools tested
*Aedes caspius*	2	1	219		1			0			1		0	0.9	0.5	220	3/64
*Aedes detritus*	1		191		3			0			0		4	0.5	0	198	1/59
*Culex pipiens*	1		57		9	1	4	52	1	1	14	1	9	2.1	4.3	141	9/71
*Aedes albopictus*			19	7	61		2	27			7		3	0	7.8	116	9/64
*Culex perexiguus*	3	1	12		0			0			0		0	25	8.3	12	4 /7

Positivity data are reported separately for *Dirofilaria immitis* and *Dirofilaria repens*. The positivity rate is calculated based on positive pools, assuming only one positive mosquito per pool

## Discussion

This study provides the first comprehensive molecular survey of *Dirofilaria* spp. in mosquito populations across Sardinia, a region where only limited information is available about its mosquito fauna [33, 34]. The findings provide new insights into the composition of local vector species, highlighting the first molecular detection of *C. perexiguus*, and their potential role in the transmission of *D. immitis* and *D. repens*. In this study, 13 native mosquito species were recorded on the island, and no invasive mosquito species were detected, except for the already well-established *Ae. albopictus*. Among these, *Ae. caspius* and *Ae. detritus* were the most abundant species, together constituting approximately 59.6% of the total mosquitoes captured, followed by *C. pipiens* (19.8%) and *Ae. albopictus* (16.6%). This pattern is consistent with the available information from the nearby island of Corsica [35], where *Ae. caspius* (79.8%) dominated the mosquito fauna, followed by *Ae. albopictus* (11.0%) and *C. pipiens* (9.2%). Notably, among minor species detected, at least 12 specimens belonging to *C. perexiguus* were collected in Oristano. Despite being described in Spain, to the best of our knowledge, the presence of *Culex perexiguus* in Italy remains controversial. Two studies refer to it as a species not yet reported in Italy [36, 37], while three other studies have reported its presence, albeit with limited robustness. In particular, Harbach et al. [30] reported having examined only a few specimens from Greece and Italy, without providing any objective supporting evidence (i.e., no bibliographic references, no information on the number of specimens, no morphological details, and no exact locality of collection). Snow and Ramsdale [31] in their review mainly referred to the former ambiguous nomenclature *Culex univittatus/perexiguus*, without distinguishing between the two species of the *C. univittatus* subgroup. Also, Robert et al. [32] reported the presence of *C. perexiguus* in Italy only anecdotally, based on the previous studies by Harbach et al. [30] and Snow and Ramsdale [31].

*Culex perexiguus* can exploit a broad range of stagnant waters, from clean to moderately polluted habitats, but it is reported to predominate in rice fields [37, 38], which exist in the province of Oristano, supporting the ecological evidence of the presence of this species in the sampled area.

*Culex pipiens* accounted for 33.3% of *D. immitis*- and 35.3% of *D. repens*-positive specimens, confirming its role as a major vector for both species in Sardinia (infection rates: 2.1% *D. immitis* and 4.3% *D. repens*) and its recognized importance in several European countries [35, 39–45]. *Aedes albopictus* also emerged as the major vector of *D. repens* in the study area, representing 52.9% of all *D. repens*-positive mosquitoes and exhibiting the highest infection rate among the tested species (7.8%). This is in line with studies from Italy and other European countries that reported *Ae. albopictus* as a natural vector for *D. repens* [35, 46]. Although *Ae. albopictus* is recognized as a competent vector also for *D. immitis* [9, 46], none of the specimens examined in this study tested positive for this parasite. This finding should be interpreted in light of both mosquito abundance and *Dirofilaria* species circulation in the sites where *Ae. albopictus* were collected. In Sant’Antioco, where the highest and most representative number of *Ae. albopictus* were captured (Table 1, Fig. 2), all positive specimens (*N* = 7) were infected by *D. repens*, while no other mosquito collected at that site tested positive for either *D. repens* or *D. immitis*. This potentially indicates a low circulation or absence of *D. immitis* in Sant’Antioco. In Sassari, the only two *Ae. albopictus* specimens were positive for *D. repens*, consistent with the higher circulation of *D. repens* (86%) than of *D. immitis* (14%) in the site (Table 4, Fig. 2). *Aedes caspius*, the most abundant mosquito species in our survey, also demonstrated its possible involvement in *Dirofilaria* transmission, as assumed elsewhere [35, 47]. In fact, this species showed a non-negligible contribution to the overall positivity of *D. repens* and, along with *C. pipiens*, represented the majority of *D. immitis*-positive mosquitoes identified in our survey. The detection of *D. immitis* in a single specimen of *Ae. detritus* further expands the list of potential vectors in Sardinia. Although this record is based on a single mosquito, the large numbers of *Ae. detritus* in the samples collected reinforce the importance of this mosquito as a potential vector, particularly in coastal habitats where flooded sites, rich in vegetation cover and high salinity, are abundant [48–51]. *Aedes detritus* is, in fact, considered a potential vector for several pathogens other than *D. immitis* [52], including arboviruses [52]. Thus, its presence in three out of five sampling sites, and its high abundance particularly in Oristano, highlights the potential public health importance of this species (Table 1). Finally, the detection of *D. immitis* and *D. repens* DNA in 4 out of 12 specimens of *C. perexiguus* in Oristano suggests the need for future investigations to evaluate its potential vector role, considering that, to the best of our knowledge, this species has never been found positive for the presence of *Dirofilaria*.

It is important to note that molecular detection of *Dirofilaria* DNA in a mosquito species does not unequivocally confirm its vector role, as it is not possible to distinguish between early stages of the parasite (microfilariae, L1, L2) and infective L3 larvae. Mosquito dissection and identification of L3 larvae would be necessary to confirm the actual transmission potential of each species in the sampled areas, especially regarding those for which evidence of a natural vector role is still lacking.

At the site level, *Dirofilaria* circulation should be interpreted considering species diversity and abundances (Fig. 2, Tables 2 and 4). Oristano province showed a high species diversity (H: 0.82) and the greatest abundance of mosquitoes collected; therefore, it was the site with the highest variety of infected species (Fig. 2). In addition, given the positivity rates observed, a high circulation of both *Dirofilaria* species can reasonably be concluded for this site. In Sant’Antioco and Sassari, only *C. pipiens* and *Ae. albopictus* were found infected, consistent with the relatively low diversity of species collected (H: 0.69 and 0.53, respectively), but with a reasonable abundance of specimens. Therefore, it can be concluded that in these two sites, *C. pipiens* and *Ae. albopictus* are the main vectors of *Dirofilaria* spp., with *D. repens* circulating more widely. Conversely, in Vallermosa, although the highest species diversity was observed (H:0.94), only one specimen of *C. pipiens* tested positive. This low prevalence is likely due to the limited number of specimens collected at this site (*N* = 29). The limited number of individuals captured also in the province of Tortolì (*N* = 33) confirms that sample size plays an important role in the likelihood of detecting positive species. In fact, although Tortolì shows a significantly lower species diversity compared to Vallermosa, the total number of specimens captured is comparable; indeed, in this site, only a single *C. pipiens* specimen tested positive. This calls for caution when extrapolating epidemiological insights from the available entomological samples from these two sites. The observed diversity at the site level suggests the influence of local characteristics on mosquito community structure and, therefore, pathogen transmission dynamics [53].

The seasonal dynamics of mosquito populations should be considered in terms of the role of different species in the *Dirofilaria* transmission risk. There is a remarkable temporal heterogeneity in the most abundant species found in the sampled areas, with *Ae. caspius* exhibiting bimodal activity peaks in June and September, while *Ae. detritus* maintained high densities from June through September. In contrast, *C. pipiens* and *Ae. albopictus* demonstrated broader seasonal activity, with *C. pipiens* present year-round and *Ae. albopictus* active from May to December. The predominance of these recognized and potential vectors in months when many tourists visit the island, often along with their pets, may represent an epidemiological risk for infection to both dogs and humans [12].

Investigating the presence of *D. repens* and *D. immitis* DNA in mosquitoes is crucial for understanding the epidemiology of dirofilariosis in Sardinia. The overall positivity rates of 1.8% for *D. repens* and 1% for *D. immitis* indicate ongoing parasite circulation within the mosquito populations. The varying infection rates across different sites suggest a spatial heterogeneity of transmission risk that is probably influenced by local factors, including the presence of infected hosts and suitable mosquito vectors. A recent survey conducted in different areas of Sardinia enrolling *N* = 467 shelter and *N* = 274 privately owned dogs, evidenced *D. immitis* and *D. repens* in 9.9% and 5.5% of tested dogs, respectively [12]. In addition, recent autochthonous human cases (Nonnis et al., in press) in Sardinia confirm the zoonotic risk. This evidence supports the large circulation of *Dirofilaria* parasites on the island, in agreement with this study.

## Conclusions

This study offers comprehensive evidence of *D. repens* and *D. immitis* circulation within Sardinian mosquito populations, revealing a diverse vector community involved in the transmission of the two parasites. Both *C. pipiens* and *Ae. albopictus* were confirmed as major vectors, with *Ae. albopictus* involved in *D. repens* transmission only. Interestingly, *Ae. caspius* and *Ae. detritus* showed non-negligible positivity rates for *Dirofilaria,* supporting the potential vector role of these species as currently hypothesized in a few other available studies. Notably, this study reports, for the first time to our knowledge, the presence of *C. perexiguus* in Italy, based on molecular data, and highlights the need for further investigation into the potential role of this species in transmitting *Dirofilaria*. The detection of *Dirofilaria* DNA in multiple mosquito species across different eco-geographical settings suggests complex transmission dynamics shaped by local environmental factors and vector ecology. Given the confirmed parasite circulation in this heterogeneous vector community, ongoing entomological and molecular surveillance is mandatory to support targeted vector control and public health strategies aimed at mitigating dirofilariosis risk for both animal and human populations in Sardinia and, possibly, in other Mediterranean countries.

## Data Availability

The datasets used and/or analysed during the current study are available from the corresponding author on reasonable request. Nucleotide sequences from this study have been deposited in GenBank under accession numbers: PX963966, PX963967, PX963968, PX963969, PX963970, PX963971, PX963972.

## Abbreviations

VBDs: Vector-borne diseases
ECDC: European Centre for Disease Control
HWD: Heartworm disease
COI: Cytochrome c oxidase subunit I
H: Shannon-Wiener Diversity Index
CI: Confidence intervals
